# Target Protein Expression on *Tetrahymena thermophila* Cell Surface Using the Signal Peptide and GPI Anchor Sequences of the Immobilization Antigen of *Cryptocaryon irritans*

**DOI:** 10.1007/s12033-023-00824-w

**Published:** 2023-07-22

**Authors:** Yuho Watanabe, Masahito Asada, Mayu Inokuchi, Maho Kotake, Tomoyoshi Yoshinaga

**Affiliations:** 1 Department of Aquatic Bioscience, Graduate School of Agricultural and Life Sciences, The University of Tokyo, 1-1-1, Yayoi, Bunkyo-ku, Tokyo, 113-8657 Japan; 2 National Research Center for Protozoan Diseases, Obihiro University of Agriculture and Veterinary Medicine, Inada-Cho, Obihiro, Hokkaido 080-8555 Japan

**Keywords:** *Cryptocaryon irritans*, *Tetrahymena thermophila*, Signal peptide, Glycosylphosphatidylinositol anchor, Parasite, Monomeric Azami-green 1

## Abstract

Cryptocaryoniasis, caused by *Cryptocaryon irritans*, is a significant threat to marine fish cultures in tropical and subtropical waters. However, controlling this disease remains a challenge. Fish infected with *C. irritans* acquires immunity; however, *C. irritans* is difficult to culture in large quantities, obstructing vaccine development using parasite cells. In this study, we established a method for expressing an arbitrary protein on the surface of *Tetrahymena thermophila*, a culturable ciliate, to develop a mimetic *C. irritans*. Fusing the signal peptide (SP) and glycosylphosphatidylinositol (GPI) anchor sequences of the immobilization antigen, a surface protein of *C. irritans*, to the fluorescent protein, monomeric Azami-green 1 (mAG1) of the stony coral *Galaxea fascicularis*, allowed protein expression on the surface and cilia of transgenic *Tetrahymena* cells. This technique may help develop transgenic *Tetrahymena* displaying parasite antigens on their cell surface, potentially contributing to the development of vaccines using “mimetic parasites”.

**Supplementary Information:**

The online version contains supplementary material available at 10.1007/s12033-023-00824-w.

## Introduction

*Cryptocaryon irritans* is an obligate parasitic ciliate of marine fishes that causes cryptocaryoniasis, also called marine white spot disease. The parasite's life cycle includes four stages: infective theront, parasitic trophont, pre-reproductive protomont, and reproductive tomont [1, 2]. Upon infection, the parasite invades the epithelial layer of the skin and gills of marine teleosts, disturbing their osmotic control and respiratory activity; severe infections often lead to mass mortalities of affected fish [3]. Cryptocaryoniasis frequently occurs in marine cage cultures and land-based aquacultures in tropical and subtropical waters, causing severe economic losses to aquaculture [4–6]. Although many studies have focused on developing control methods such as therapeutic drugs and vaccines, efficient treatments for infections caused by *C. irritans* have not been developed, especially for food fishes in net cage culture [5]. Oral administration of lysozyme chloride appears to be an effective treatment of *C. irritans* and is approved for use on food fish in Japan (https://www.maff.go.jp/j/syouan/suisan/suisan_yobo/attach/pdf/fishmed-24.pdf). However, the treatment alone is not enough to control *C. irritans* infection. Therefore, effective chemotherapies and vaccines against these parasitic diseases are required.

Previous studies have demonstrated that fishes infected with *C. irritans* produce antibodies against the parasite proteins and acquire protective immunity [7, 8]. Immunization with formalin-killed theronts can induce protective immunity against parasitic infections [9]. Therefore, immunization with *C. irritans* whole cells may be an effective way to control cryptocaryoniasis. However, because in vitro culture methods for parasite propagation are unavailable, the parasite is mainly propagated using live fish [4]. Thus, a mass culture of *C. irritans* for commercial vaccine development is costly and impractical.

Several studies have used a culturable ciliate *Tetrahymena thermophilla* as a model organism [10], and the protocol for its transformation has been well established [11, 12]. Additionally, a recombinant protein of *C. irritans* was successfully expressed using the *Tetrahymena* expression system [13]. Thus, using *Tetrahymena* cells may contribute to developing a vaccine against cryptocaryoniasis. Other recent studies have identified several infection-protective antigens against *C. irritans* infection [14–16]. Moreover, *T. thermophila* shares a cross-reactive antigen with *C. irritans*, and vaccination with *T. thermophila* induces protective immunity against *C. irritans* infection, thereby demonstrating cross-protection [17]. The development of transgenic *Tetrahymena* cells expressing these infection-protective antigens (“mimetic parasites”) can lead to more effective vaccines.

However, considering its practical application in aquaculture farms, inoculating fish with live transgenic organisms as a vaccine is challenging; therefore, inactivation with formalin is necessary. Antibodies produced by inoculating formalin-inactivated bacteria mainly target cell surface antigens [18, 19]. Thus, by exposing antigenic proteins on the surface of *Tetrahymena* cells, antigens are easily recognized by host cells even after inactivation, and effective immunity against parasites can be induced.

Some ciliates, such as *Tetrahymena thermophila*, *Paramecium aurelia*, and *I. multifiliis*, have immobilization antigens (i-antigens) on the cell surface [20, 21]. Structurally, i-antigens contain a glycosylphosphatidylinositol (GPI) modification at their C-termini, which anchors them to plasma and surface membranes [22, 23]. The i-antigens elicit the production of antibodies that immobilize ciliates and are considered potential vaccine candidates against ciliate infections [24–28]. It is expected that immunization of fish with *Tetrahymena* cells that express parasite antigens on their cell surface as well as i-antigens, will induce strong antibody production against the target parasite antigens.

In this study, we established a method for expressing an arbitrary protein on the surface of *Tetrahymena* cells using the sequences of a signal peptide and the GPI anchor of the i-antigen of *C. irritans* to develop mimetic *C. irritans*.

## Materials and Methods

### Parasite

*Cryptocaryon irritans* (isolate UT2) was originally isolated from an ornamental scalpel saw tail, *Prionurus scalprum*, purchased from a local pet shop in Tokyo [15]. The parasite isolate was passaged and propagated in seawater-adapted black mollies (*Poecilia* sp.; mean body length, 3–4 cm) in our laboratory, as described by Watanabe et al. [29]. Theronts, protomonts, and tomonts obtained from routine propagation were used in the experiments.

### cDNA Cloning of an i-Antigen Gene of UT2

Total RNA was extracted from trophonts (1000 cells) using a NucleoSpin RNA Plus Kit (Macherey–Nagel, Dürren, Germany), treated with DNase I (Takara Bio, Shiga, Japan) to remove genomic DNA, and purified using a NucleoSpin RNA Clean-up Kit (Macherey–Nagel). Purified RNA (100 ng) was reverse transcribed using the ReverTra Ace qPCR RT Master Mix with gDNA Remover (Toyobo, Osaka, Japan). Reverse transcription (RT)-PCR was performed using KOD One PCR Master Mix (Toyobo, Osaka, Japan) with primers for the i-antigen gene [14] (#1, #2, Table 1), following the program as 40 cycles of 98 °C for 10 s, 55 °C for 5 s, and 68 °C for 5 s.

**Table 1:** Primers used in this study

	Primers		Sequence 5′–3′	References
#1	i-antigen	F	YMGCWGATTGGAMHGGWAC	Watanabe et al. [14]
#2	i-antigen	R	AGTACTWAATTCATGAATAT	
#3	i-antigen_5′-RACE		AGTTACACAAGCACCAGAGGCTATTGTGC	This study (RACE)
#4	i-antigen_3′-RACE		ATGGCATGGTCTGACAATGCTAGTGC	This study (RACE)
#5	MTT1-pro_FW	F	CCCGGATCCGCGGCCGCAGACAATTTATTTCTA	This study (In-fusion)
#6	MTT1-pro_RV	R	AAAAATTTTATTCATTATTTTAAGTTTAGTATT ATTATTTATTTTATTAGAGC	This study (In-fusion)
#7	Extr.mAG1_FW	F	ATGAATAAAATTTTTATTGCTTTATTAGTAGTACT ATTAGCAGTATCAACATAGACAGCAGAACAGAA ATTAATAAGTGAAGAGGATTTGGAGCAAAAGTT GATTTCAGAGGAAGACTTAAGTGGAGGAGGTG GTTCTATGGTGAGTGTGATTAAACCAG	This study (In-fusion)
#8	Extr.mAG1_RV	R	TTTAATTTAAGGATCTCATTTGAATAAGAGGCCA AGAGTTAATAAGATCATAATGCTTAATCCTTAAA CTAATTTAATAGCATTTGAACCTGCTTTTGACTT GGCCTGACTCGGCAGCATAG	This study (In-fusion)
#9	BTU2-ter_FW	F	GATCCTTAAATTAAAAATTCAATA	This study (In-fusion)
#10	BTU2-ter_RV	R	GAGAATTCCGCGGCCGCTGCATTTTTCCAG TAAAAATTTG	This study (In-fusion)

In addition, the complete cDNA sequence of the UT2 i-antigen was determined using the SMART rapid amplification of cDNA ends (RACE) cDNA Amplification Kit (Clontech, Palo Alto, CA, USA) according to the manufacturer’s instructions. The gene-specific primers used for RACE-PCR are listed in Table 1 (#3 and #4). Each amplified RACE-PCR product was cloned into the pGEM-T Easy Vector (Promega, Madison, WI, USA) and sequenced by Eurofins Genomics K.K. (Tokyo, Japan).

### Sequence Analysis of UT2 i-Antigen

The ORF of the UT2 i-antigen was searched by “Translate” (https://web.expasy.org/translate/). The theoretical molecular weight was predicted using the Compute pI/Mw tool (http://web.expasy.org/compute_pi/), and the signal peptides were predicted using SignalP-6.0 (https://services.healthtech.dtu.dk/services/SignalP-6.0/). GPI-anchored protein and ω-site were predicted using PredGPI (https://busca.biocomp.unibo.it/predgpi/). Sequence similarity with other known proteins was analyzed using the protein–protein BLAST program on the NCBI server (https://blast.ncbi.nlm.nih.gov/Blast.cgi).

### Plasmid Construction

**Fig. 1:**
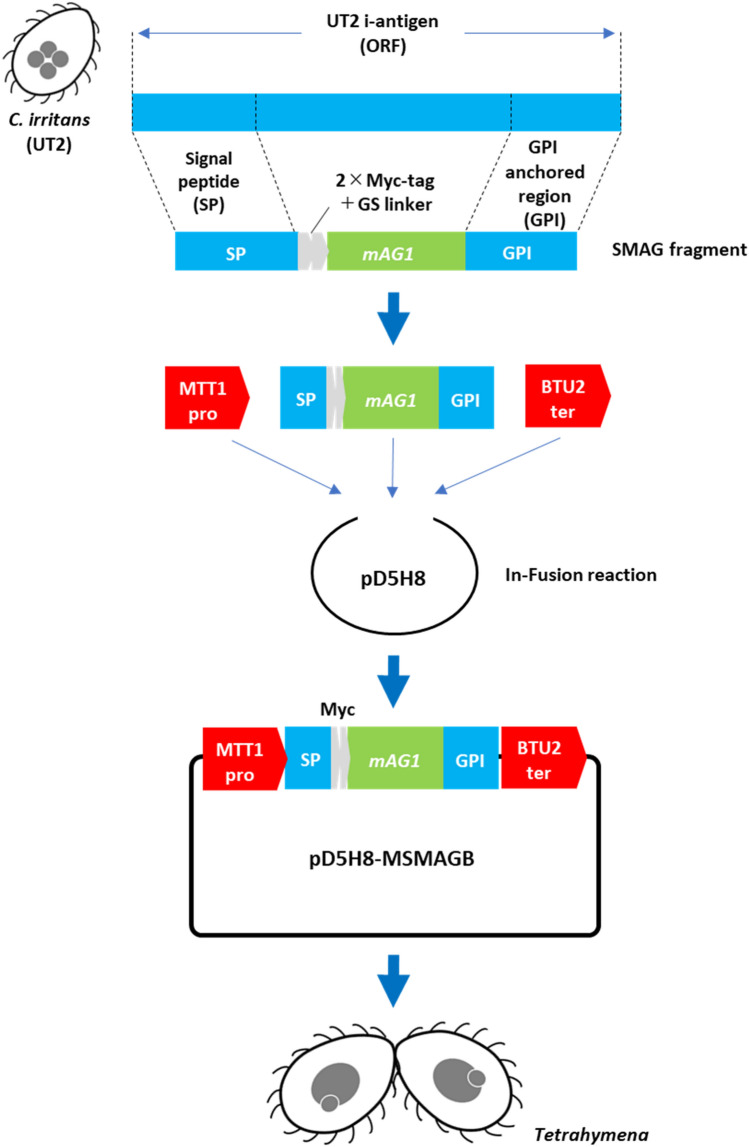
Production of pD5H8-MSMAGB plasmid and generation of transgenic *Tetrahymena* cells expressing for r-SP-Myc-mAG1-GPI

The procedure for plasmid construction is shown in Fig. 1. Using primers containing the signal peptide and GPI anchor sequence of the i-antigen predicted by the above analysis (#7 and #8, Table 1), SP-Myc-mAG1-GPI (SMAG) fragment was obtained by fusing the signal peptide sequence, Myc tag, and GPI anchor sequence to the fluorescent protein monomeric Azami-green 1 (mAG1) gene from pmAG1-MN (MEDICAL & BIOLOGICAL LABORATORIES CO., LTD., Tokyo, Japan). The cadmium-inducible MTT1 promoter [30] and the beta-tubulin 2 BTU2 terminator [30] were amplified from the Cas9 expression vector pC9T (*Tetrahymena* Stock Center, Cornell University) [31] using the primer pairs #5 and #6, and #9 and #10, respectively. The SMAG fragment was cloned into a unique NotI site in the ribosomal DNA vector pD5H8 [32] using an In-Fusion HD Cloning Kit (Takara Bio), along with the MTT1 promoter and BTU2 terminator fragments. The final plasmid, pD5H8- MTT1-SP-Myc-mAG1-BTU2 (MSMAGB), was sequenced (Eurofins Genomics, Tokyo, Japan) to confirm the correct insertion of the target sequences. The pD5H8 vector contains *Tetrahymena* MIC rDNA with a paromomycin-resistant allele of the 17S rRNA [33]. The rDNA allele has a mutation conferring resistance to paromomycin and other sequence differences conferring a replication advantage, allowing the selection of transformants and complete replacement of endogenous rDNA [32, 34]. Following the transformation of *E. coli* (DH5α), the plasmid DNA (pD5H8-MSMAGB) was isolated and introduced into *T. thermophila*, as described below.

### *Tetrahymena* Cell Culture and Transformation

Cell culture and transformation were performed with slight modifications from previous studies [13, 35]. *T. thermophila* strains CU427 (mating type 6) and CU428 (mating type 7) were obtained from the *Tetrahymena* Stock Center, and cultured separately in NEFF medium (0.25% protease peptone, 0.25% yeast extract, 0.5% glucose, and 33 µM FeCl_3_) at 30 °C for 24 h with shaking (80 rpm). At a density of ~ 5 × 10^5^ cells/mL, 1.0 × 10^7^ cells of each strain were centrifuged at 700 × *g* for 3 min. The medium was discarded, and cells were suspended in 10 mL of 10 mM Tris–HCl (pH 7.5) and incubated at 30 °C for 24 h with shaking (80 rpm). The cells were counted and adjusted to a final density of 2 × 10^5^ cells/mL. Equal numbers of cells from each culture were mixed and incubated for 10 h. Cells were examined microscopically to ensure that more than 80% of the cells had undergone mating. After conjugation, the cells were transferred to 50 mL tubes and gently washed twice with 10 mM HEPES (pH 7.5). The cells were concentrated and resuspended in HEPES at 3 × 10^7^ cells/mL density. Cell suspension (125 µL) and pD5H8-MSMAGB plasmid (30 µg in 125 µL of HEPES) were mixed and transferred into a 0.2 cm electroporation cuvette. Electroporation was performed by applying a constant voltage (250 V) for 2.8 ms using the MicroPulser Electroporator (Bio-Rad Laboratories, Hercules, CA, USA). The cuvettes were rested for 1 min, and the cells were resuspended in 10 mL NEFF medium. Then, 100 µL/well NEFF medium was transferred to a 96-well plate. After 18 h post-electroporation, 100 µL of NEFF containing 200 µg/mL paromomycin sulfate was added to each well, resulting in a final concentration of 100 µg/mL paromomycin sulfate. The plates were incubated at 30 °C for 4 days. Wells containing paromomycin-resistant cells were successfully transferred into NEFF medium with 150, 200, and 400 µg/mL paromomycin to select the final transformed cell lines.

### Expression of mAG1 Fused with a Signal Peptide and GPI Anchor Motif

The MTT1 promoter of *T. thermophila* regulates the expression of mAG1 [30]. This promoter system was induced by adding cadmium ions to the culture medium. Transgenic *Tetrahymena* cells were cultured in NEFF medium at a density of 1.0 × 10^5^ cells/mL. For inducing the cells, CdCl_2_ was added to a final concentration of 10 µM, the cells were induced by incubating at 30 °C with agitation for 6 h. The cells were harvested and observed under a fluorescence microscope (BX60; Olympus, Tokyo, Japan) to confirm recombinant-SP-Myc-mAG1-GPI expression and signal localization.

### Indirect Immunofluorescence Antibody Test (IFAT)

IFAT was performed on air-dried transformed cells fixed in a 1:1 acetone: methanol mixture for 10 min at − 20 °C. Cells were immunostained with rabbit anti-Myc polyclonal antibody (16286-1-AP; Proteintech, Rosemont, IL, USA) or rabbit anti-mAG1 polyclonal antibody (PM052M; MEDICAL & BIOLOGICAL LABORATORIES CO., LTD.) at 1:100 dilution in PBS supplemented with 0.1% Tween 20 (PBS-T) and incubated at 37 °C for 60 min. The cells were then incubated with Alexa fluor 488-conjugated donkey anti-rabbit IgG antibody (1:200) at 37 °C for 30 min. For staining the nuclei, the cells were incubated with 1 μg/mL Hoechst 33,342 solution at 37 °C for 20 min. The cells were examined using fluorescence and confocal laser-scanning microscopes (C1, Nikon, Tokyo, Japan).

### Protein Extraction

*Tetrahymena* cells (2.0 × 10^5^ cells) that expressed r-SP-Myc-mAG1-GPI were centrifuged at 2000×*g* for 10 min at 20 °C and washed thrice with 10 mM Tris–HCl (pH 7.5). The pellet was resuspended in 50 μL of PBS containing protease inhibitor cocktail (cOmplete Mini, Roche, Basel, Switzerland) and freeze-thawed thrice. The resultant suspension was vortexed for 15 s and centrifuged at 20,000×*g* for 30 min at 4 °C. The supernatant containing cytosolic proteins (cytosolic fraction) was collected.

The pellet (membrane fraction) was washed thrice with PBS to remove cytosolic proteins and then resuspended in the PBS containing 1% Triton X-100, 2% sodium dodecyl sulfate (SDS), and a protease inhibitor cocktail. The resultant suspension was vortexed for 15 s and centrifuged at 20,000×*g* for 30 min at 4 °C. The supernatant containing membrane proteins was collected.

Ciliary proteins were obtained using the calcium shock method described by Motokawa et al. [36]. Briefly, ciliates (1.0 × 10^7^ cells) were pelleted in the same manner as for the preparation of the cytosolic fraction. The pelleted cells were resuspended in 1 mL of ice-cold solution A (10 mM EDTA, 50 mM sodium acetate; pH 5.0) and kept on ice for 30 s. One milliliter of ice-cold sterile distilled water was added, and the suspension was gently mixed by inversion for 90 s and kept on ice for 120 s. Fifty microliters of ice-cold 0.2 M CaCl_2_ solution was added, and the mixture was allowed to stand on ice for 3 min and then stirred gently for 1 min. Deciliation was confirmed by light microscopy, and the resultant suspension was centrifuged at 400×*g* for 3 min at 4 °C. The supernatant was collected and centrifuged twice in the same manner. The cilia in the supernatant were pelleted by centrifugation at 20,000×*g* for 15 min at 4 °C. The ciliary pellets were resuspended in 50 µL of PBS containing 1% Triton X-100, 2% SDS, and a protease inhibitor cocktail. The resultant suspension was vortexed for 15 s and centrifuged at 20,000×*g* for 30 min at 4 °C. The supernatant containing ciliary proteins (ciliary fraction) was collected. The concentration of each protein solution was estimated using a bicinchoninic acid (BCA) protein assay kit (Takara Bio) following the manufacturer's protocol.

### SDS–Polyacrylamide Gel Electrophoresis (PAGE) and Western Blotting

All proteins were analyzed using SDS-PAGE under reducing conditions, followed by western blotting, as described by Watanabe et al. [14]. Protein solutions (6.5 μL) containing 2.5 μg of the protein were mixed with 2.5 μL of 4X NuPAGE™ LDS sample buffer (Thermo Fisher Scientific, Waltham, MA, USA) and 1.0 μL of 10 × NuPAGE™ sample reducing agent (Thermo Fisher Scientific) and then heated at 70 °C for 10 min. The samples (2.5 μg extracted proteins) were loaded on 12% acrylamide gels and electrophoresed at 200 V for 45 min (SDS Mini-PROTEAN Tetra System; Bio-Rad). The SDS gels were either stained with Coomassie Brilliant Blue or transferred to nitrocellulose membranes using a semi-dry transfer system (WSE-4020; ATTO, Tokyo, Japan) at 153 mA for 30 min. The membrane was blocked using 5% non-fat milk for 2 h at 25 °C. The membrane was washed thrice with PBS-T and incubated overnight at 4 °C, in 5% non-fat milk containing the rabbit anti-Myc polyclonal antibody (1:2000 dilution) or rabbit anti-mAG1 polyclonal antibody (1:2000) as the primary antibody. The membrane was washed with PBS-T and incubated with horse radish peroxidase (HRP)-conjugated anti-rabbit IgG (1:10,000; Jackson Immuno Research Laboratories, Inc., West Grove, PA, USA) as a secondary antibody with gentle shaking for 1 h at 25 °C, and washed again. Chromogenic detection was performed using Ez West Blue (ATTO) as the substrate for HRP detection.

### Immobilization/Aggregation Assay

After incubating transgenic *Tetrahymena* cells with 10 μM CdCl_2_ for 6 h, they were transferred to a 96-well microtiter plate and exposed to rabbit anti-mAG1 polyclonal antibody at a dilution of 1:100. Uninduced cells were used as negative controls. Subsequently, digital images were captured using an inverted microscope (Olympus IX71, Tokyo, Japan).

### Ethics Statement

Although fishes are not covered by national or university animal ethics protocols, care was taken to avoid unnecessary pain while handling the experimental fish, and 2-phenoxy ethanol was used for analgesia and euthanasia, considering animal ethics.

## Results

### cDNA Cloning of an i-Antigen Gene of UT2

**Fig. 2:**
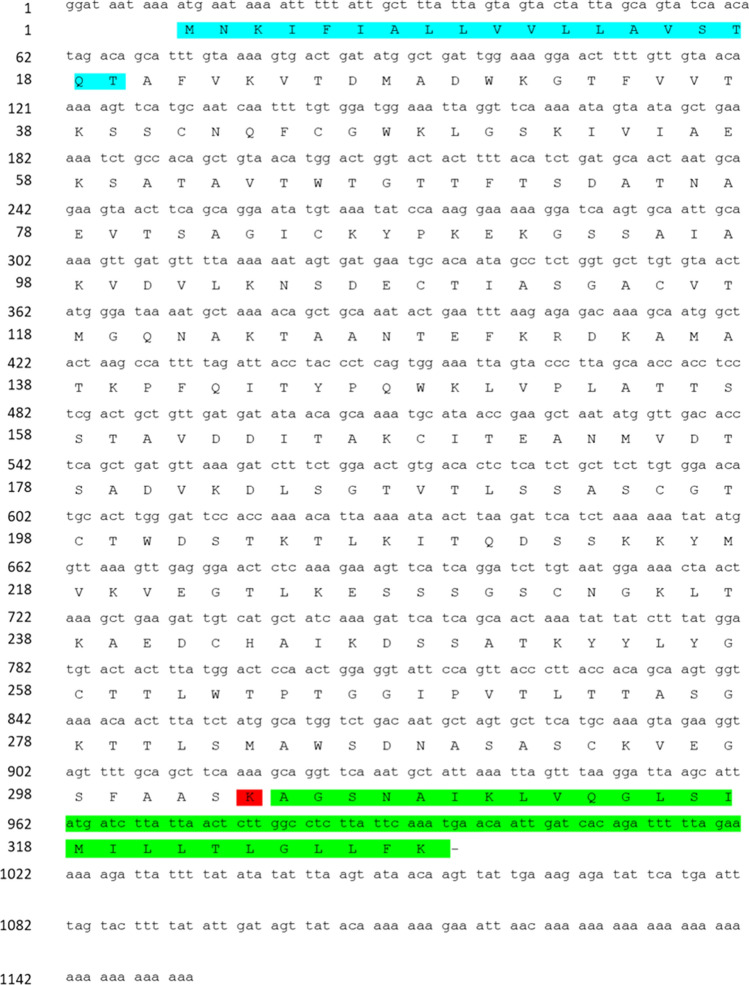
Characteristics of the UT2 i-antigen gene. The signal peptide and GPI-anchored region are indicated by black letters on a blue and green background, respectively. A red background indicates the potential ω-site

The RACE-PCR identified the cDNA sequence of the UT2 i-antigen gene (Fig. 2) (GenBank no. LC759645). The identified cDNA consisted of 1153 bp, and the first ATG at positions 11–13 was probably the initiation codon. The TGA at positions 995–997 was assigned as the termination codon. Thus, an open reading frame (ORF) of 986 bp was predicted (Fig. 2). The open reading frame encodes 328 amino acids, and 19 amino acids at the N-terminal side were predicted as the signal peptide by the SignalP-6.0 server. Based on this prediction, the molecular masses and the isoelectric point of mature UT2 i-antigen were estimated as 32,508.15 and 8.81 Da, respectively. Additionally, amino acid sequence analysis revealed that the UT2 i-antigen had a putative GPI anchor at its C-terminus and a potential ω-(cleavage) site at the 303^rd^ residue. A protein–protein BLAST search in the NCBI revealed that the UT2 i-antigen amino acid sequence was 75.17% identical to the *C. irritans* i-antigen (GenBank no. AEE39297.1), isolated from marine fish in Taiwan.

### Transgenic *Tetrahymena* Expressing mAG1 Fused with a Signal Peptide and GPI Anchor Motif

**Fig. 3:**
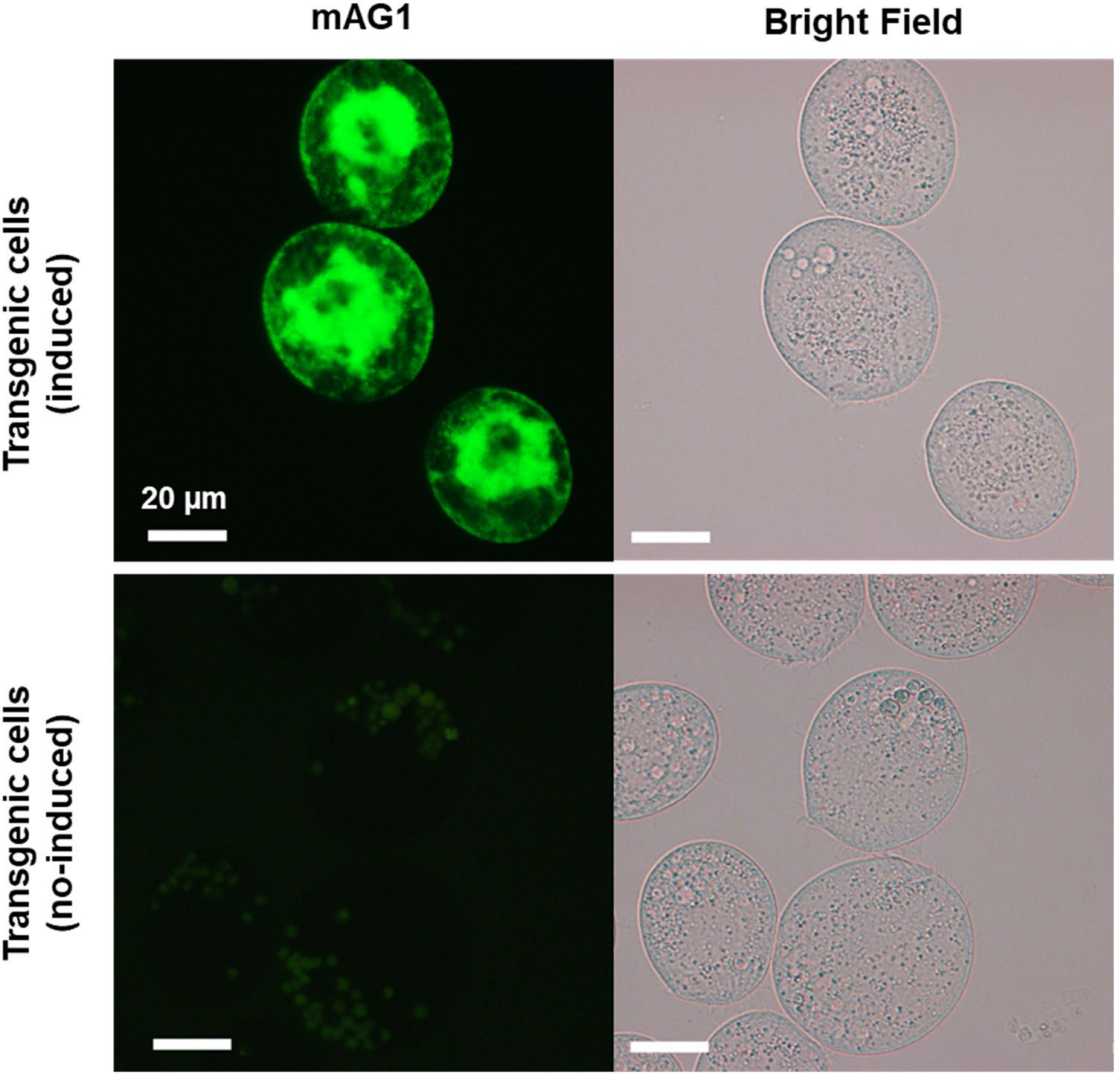
Localization of r-SP-Myc-mAG1-GPI in live transgenic *Tetrahymena* cells. Scale bar = 20 μm

Several positive clones were obtained after transformation and paromomycin selection. Target protein expression was induced by CdCl_2_ treatment in these cells, and similar r-SP-Myc-mAG1-GPI signals were detected in each clone using fluorescence microscopy. In particular, r-SP-Myc-mAG1-GPI signals were detected abundantly on the membrane and in the intracellular regions of the transgenic *Tetrahymena* cell (Fig. 3). However, no signal of r-SP-Myc-mAG1-GPI was observed in the cilia in live cell observations.

### Localization of mAG1 Fused with the Signal Peptide and GPI Anchor Motif

**Fig. 4:**
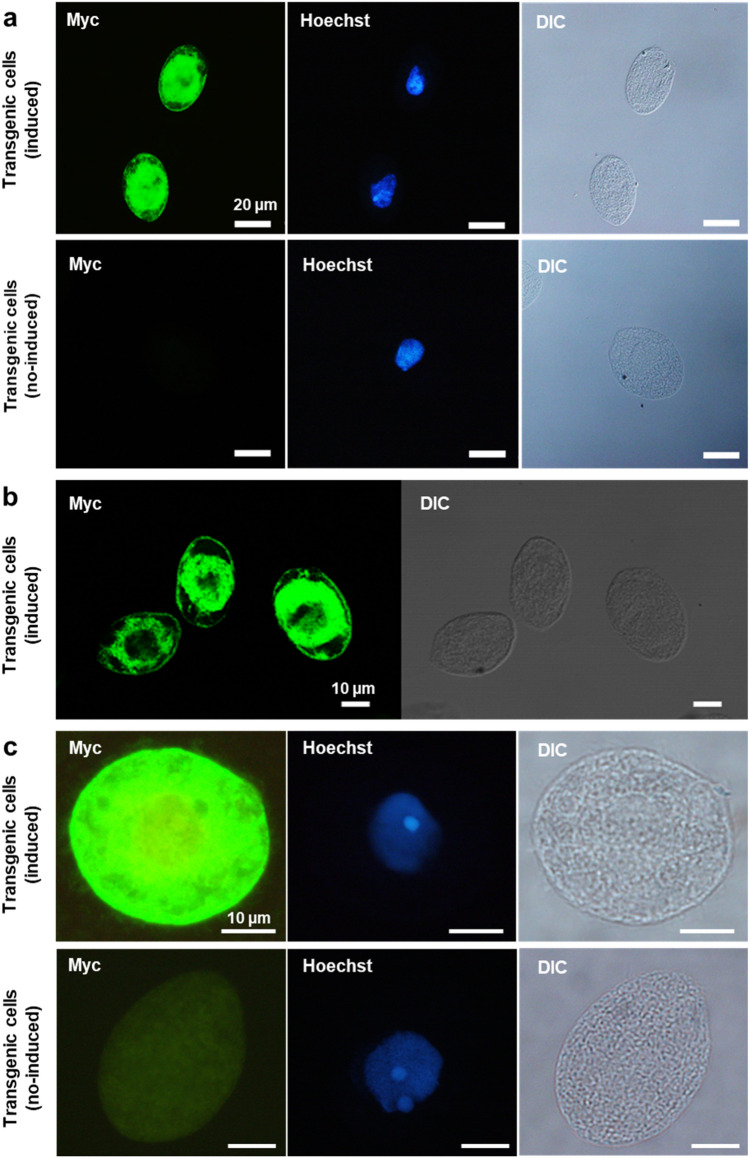
Immunofluorescence antibody test of transgenic *Tetrahymena* cells (pD5H8-MSMAGB) using the anti-Myc antibody. **a** Immunofluorescence using anti-Myc antibody showing transgenic *Tetrahymena* cells; scale bar = 20 μm. **b** Confocal laser-scanning microscopy images of transgenic *Tetrahymena* cells detected with anti-Myc antibody; scale bar = 10 μm. **c** Immunofluorescence using anti-Myc antibody showing transgenic *Tetrahymena* cells at a longer exposure time. Scale bar = 10 μm

IFAT using anti-Myc antibody revealed intense r-SP-Myc-mAG1-GPI signals in the cell membrane and around the nucleus, similar to the r-SP-Myc-mAG1-GPI signals in living cells (Fig. 4a). The cellular localization of the r-SP-Myc-mAG1-GPI protein was further investigated using confocal laser-scanning microscopy, and intense fluorescence was detected in the membrane and intracellular regions (Fig. 4b). When images were acquired at a higher exposure time, the r-SP-Myc-mAG1-GPI signals were detected in the cilia also, but were weaker than the signal detected in the membrane (Fig. 4c). In uninduced transgenic *Tetrahymena* cells, no signal was observed in the cilia. In addition, IFAT using anti-mAG1 antibody showed strong r-SP-Myc-mAG1-GPI signals in the cell membrane and the intracellular regions (Fig. S1a). The r-SP-Myc-mAG1-GPI signals in the cilia were clearer when the anti-mAG1 antibody was used in IFAT (Fig. S1b).

### SDS-PAGE and Western Blotting

**Fig. 5:**
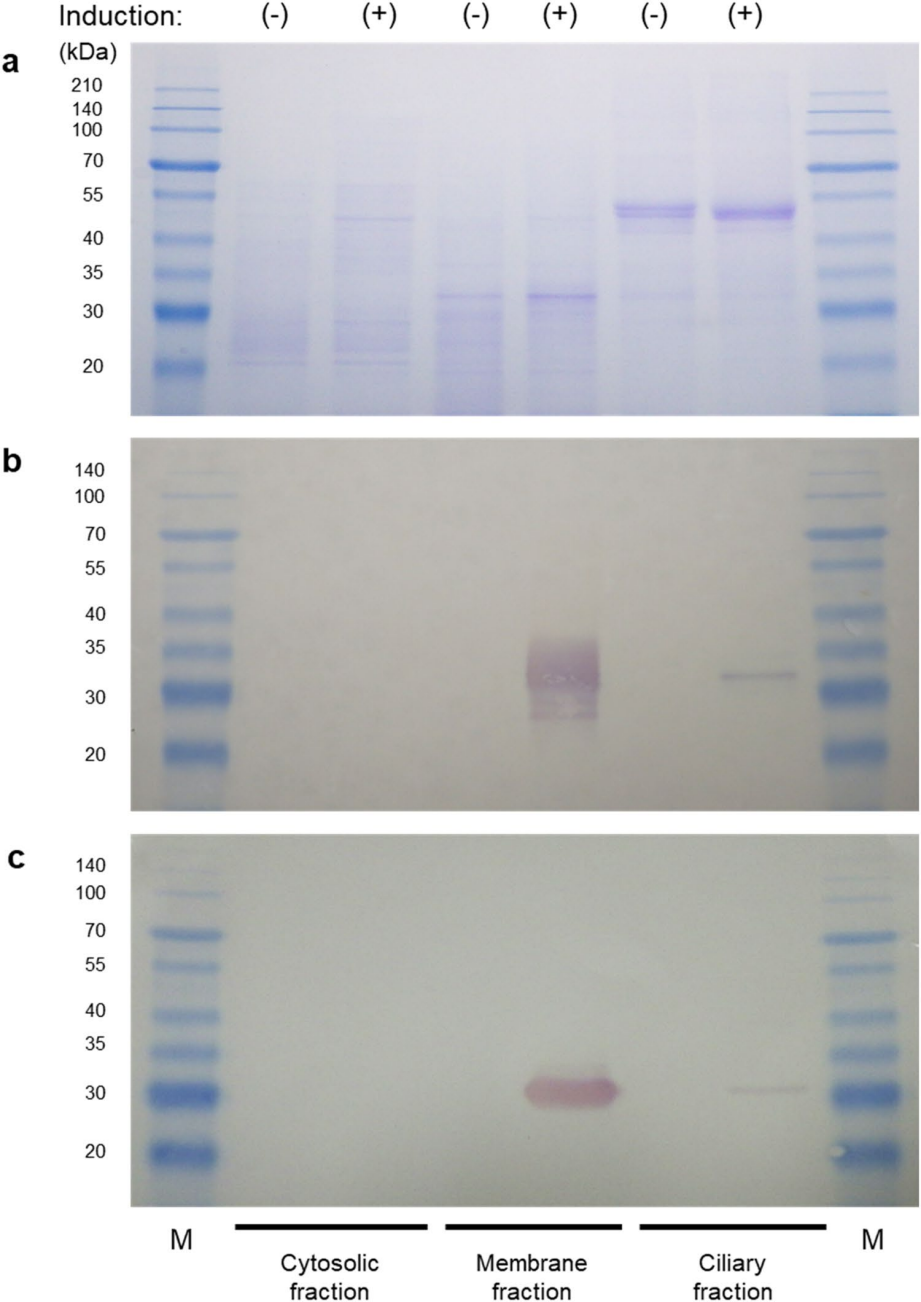
Protein expression in transgenic *Tetrahymena* cells (pD5H8-MSMAGB). **a** SDS-PAGE of transgenic *Tetrahymena* lysates with and without induction of expression with CdCl2. **b** Western blot of the lysates using the anti-Myc antibody. **c** Western blot of the lysates using the anti-mAG1 antibody. M: molecular weight maker

The SDS-PAGE result confirmed equal loading (Fig. 5a). In western blotting using the anti-Myc antibody, r-SP-Myc-mAG1-GPI protein was detected primarily in the membrane fraction and smaller amounts in the ciliary protein fraction (Fig. 5b). Immunoblotting using anti-mAG1 antibody also yielded a similar pattern (Fig. 5c). The r-SP-Myc-mAG1-GPI protein was detected at a molecular weight of 31.0 kDa in both western blotting experiments, which was close to the molecular weights predicted by its amino acid sequence (28.8 kDa). The difference between the observed and predicted molecular weights may be due to post-translational modification of the protein within the *Tetrahymena* cells. The thick band may correspond to the intracellular precursor of r-SP-Myc-mAG1-GPI protein, or membrane-bound protein attached to the signal peptide, or a non-cleaved GPI anchor signal.

### Immobilization/Aggregation Assay

**Fig. 6:**
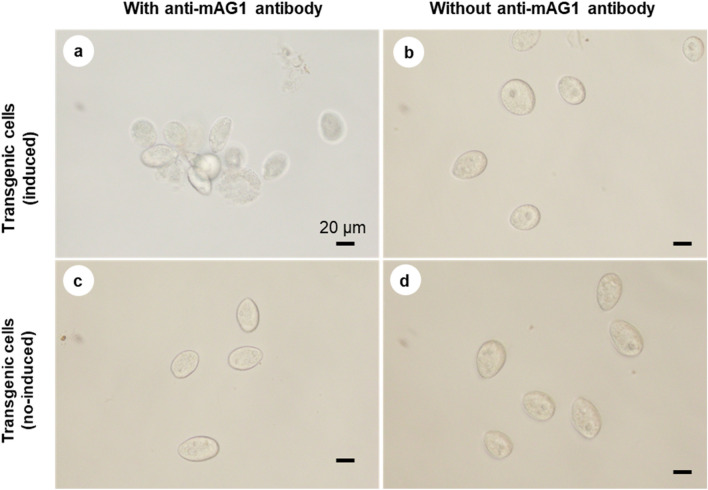
Immobilization/aggregation assay with transgenic *Tetrahymena* cells (pD5H8-MSMAGB). **a** Expression-induced transgenic *Tetrahymena* cells incubated with the anti-mAG1 antibody. **b** Expression-induced transgenic *Tetrahymena* cells incubated without the anti-mAG1 antibody. **c** Uninduced transgenic *Tetrahymena* cells incubated with the anti-mAG1 antibody. **d** Uninduced transgenic *Tetrahymena* cells incubated without the anti-mAG1 antibody. Scale bar = 20 μm

When transgenic *Tetrahymena* cells with cadmium chloride-induced expression of r-SP-Myc-mAG1-GPI were incubated with the anti-mAG1 antibody, immobilized and/or aggregated cells were observed within 30 min of incubation (Fig. 6a). In contrast, when uninduced transgenic *Tetrahymena* cells were incubated with the anti-mAG1 antibody, no immobilization or aggregation was observed (Fig. 6c). In the absence of the mAG1 antibody, immobilization or aggregation was not observed in both induced and uninduced cells (Fig. 6b, d).

## Discussion

*Tetrahymena thermophila* is an ideal candidate for vaccine production, especially against parasitic ciliates, because it is nonpathogenic, has a short generation time, and can be cultured at high cell density in inexpensive media [37]. Furthermore, *T. thermophila* has a cross-reactive antigen with *C. irritans*, and vaccination with *T. thermophila* provides cross-reactive protective immunity against *C. irritans* infection [17]. Thus, transgenic *Tetrahymena* cells expressing *C. irritans* infection-protective antigens on their surface can contribute to developing a vaccine against cryptocaryoniasis.

This study showed that fusing the signal peptide and GPI anchor sequences of the i-antigen to mAG1 allowed protein expression on the cell membrane of transgenic *Tetrahymena* cells. Western blotting, IFAT, and immobilization assay suggested that mAG1 (r-SP-Myc-mAG1-GPI) was expressed on the cilia surface also. Although mAG1 was used as a model in this study, this method can be applied to express various proteins on the *Tetrahymena* cell surface. Furthermore, this technique is expected to result in the generation of transgenic *Tetrahymena* that expresses parasite antigens on their cell surfaces, which may serve as vaccine development platform using "mimetic parasites".

Furthermore, this study strongly suggests that *C. irritans* i-antigen signal peptides and GPI anchor sequences contribute to the trafficking of the protein to the cell membrane. Previous studies have reported the presence of i-antigen on the cell surface of *C. irritans* [26, 38]; however, the region/sequence required for the protein expression on the cell surface was unknown. This study is the first to show that the signal peptide and GPI anchor sequence of i-antigen allow its expression (i.e., of the recombinant protein) on the cell surface. Additionally, when recombinant mAG1 was expressed without fusing the signal peptide and GPI anchor sequence in our preliminary experiments (Materials and methods for the construction of the plasmid used in the preliminary experiment are described in the Supplementary material, Fig. S2), the r-mAG1 signal was detected throughout the cytoplasm in IFAT and immunoblotting, using the anti-mAG1 antibody (Figs. S3, S4). These findings indicate that the signal peptide and GPI anchor sequence help translocate ciliate proteins to the cell surface and cilia. However, the r-SP-Myc-mAG1-GPI was detected in cilia by IFAT but not by live cell observation. This may be because the expression of the r-SP-Myc-mAG1-GPI in cilia was relatively low and could not be detected without amplifying the signal as in IFAT using a secondary antibody. Furthermore, compared to previous studies using transgenic *Tetrahymena* expressing *Ichthyophthirius multifiliis* i-antigen alone [39], the transgenic *Tetrahymena* cells produced in this study expressed less target protein on the cilia, and their aggregation reaction to the anti-mAG1 antibody was slightly weaker. Therefore, factors other than signal peptides and GPI anchor sequences might be involved in i-antigen translocation to the surface membrane or cilia. Although this was not clear from the present study, the accumulation of such findings will elucidate the factors involved in the subcellular localization of proteins in protozoa, including ciliates, and contribute to improving the accuracy for predicting the localization of proteins from their amino acid sequences in the future. Furthermore, this study contributes to generating a model system that can express any protein at a targeted location, enabling the development of better vaccines against *C. irritans* and other parasitic ciliates.

## Conclusions

We established a method for expressing an arbitrary protein on the surface of *Tetrahymena* cells using a signal peptide and GPI anchor sequences of immobilization antigen of *Cryptocaryon irritans*. Although further studies are needed to determine the factors involved in the localization of cell surface and ciliary proteins, this technique may help generate immunogenic mimics of parasitic ciliates, including *C. irritans*, which can contribute to developing vaccines and other products.

### Supplementary Information

Below is the link to the electronic supplementary material.

Supplementary file1 (DOCX 3647 KB)
