# Supplementary material for: Target Protein Expression on Tetrahymena thermophila Cell Surface Using the Signal Peptide and GPI Anchor Sequences of the Immobilization Antigen of Cryptocaryon irritans
Source: Mol Biotechnol. 2023 Jul 22;66(8):1907–18. doi: 10.1007/s12033-023-00824-w (PMC11282128; doi:10.1007/s12033-023-00824-w)
Supplement: Supplementary file 1 — Supplementary file1 (DOCX 3647 KB) [file 12033_2023_824_MOESM1_ESM.docx]

**Target protein expression on *Tetrahymena thermophila* cell surface using the signal peptide and GPI anchor sequences of the immobilization antigen of *Cryptocaryon irritans***

Yuho Watanabe^1,^*, Masahito Asada^2^, Mayu Inokuchi^1^, Maho Kotake^1^, and Tomoyoshi Yoshinaga^1^

^1^Department of Aquatic Bioscience, Graduate School of Agricultural and Life Sciences, The University of Tokyo, 1-1-1, Yayoi, Bunkyo-ku, Tokyo 113-8657, Japan

^2^National Research Center for Protozoan Diseases, Obihiro University of Agriculture and Veterinary Medicine, Inada-cho, Obihiro, Hokkaido 080-8555, Japan

*Corresponding author

Department of Aquatic Bioscience, Graduate School of Agricultural and Life Sciences, The University of Tokyo, 1-1-1, Yayoi, Bunkyo-ku, Tokyo 113-8657, Japan

Tel/Fax: +81-3-5841-5283

E-mail: yuhowatanabe@g.ecc.u-tokyo.ac.jp


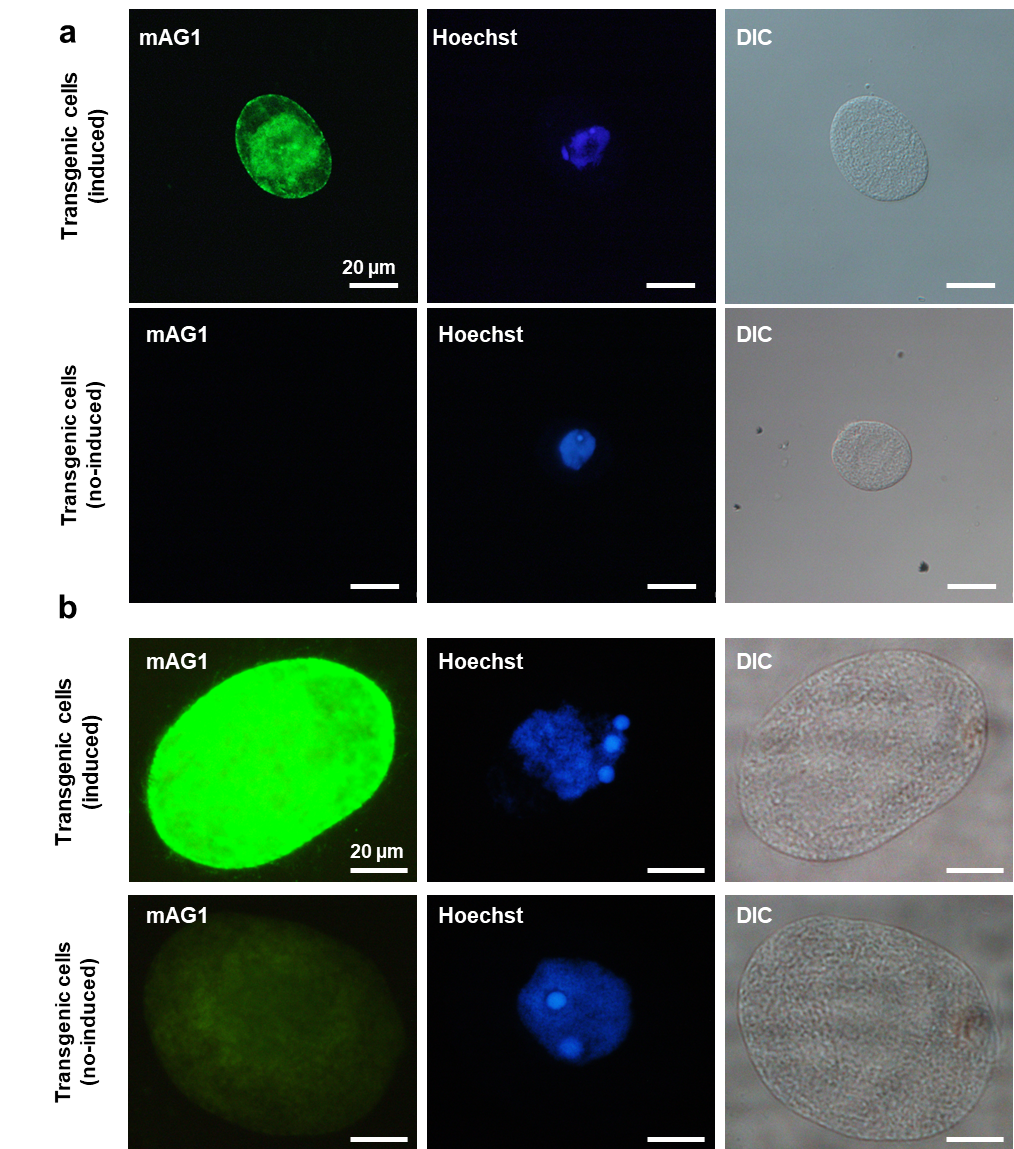


**Fig. S1** Immunofluorescence antibody test of transgenic *Tetrahymena* cells (pD5H8-MSMAGB) using the anti-mAG1 antibody. (a) Fluorescence microscopy images of transgenic *Tetrahymena* cells at IFAT with the anti-mAG1 antibody. Scale bar = 20 μm. (c) Fluorescence microscopy images of transgenic *Tetrahymena* cells at IFAT with the anti-mAG1 antibody, with a higher exposure time. Scale bar = 10 μm.

**pD5H8-MAB plasmid construction**

The procedure for pD5H8-MAB plasmid construction is shown in Figure S1. Tthe fluorescent protein monomeric Azami-green 1 (mAG1) gene was obtained from pmAG1-MN (MEDICAL & BIOLOGICAL LABORATORIES CO., LTD., Tokyo, Japan) using the primer pair #S1 and #S2 (Table S1). The cadmium-inducible MTT1 promoter [**30**] and the beta tubulin 2 BTU2 terminator [30] were amplified from the Cas9 expression vector pC9T [**31**] (*Tetrahymena* Stock Center, Cornell University) using the primer pairs #S3 and #S4, and #S5 and #S6, respectively. The mAG1, MTT1 promoter, and BTU2 terminator fragments were cloned into a unique NotI site in the ribosomal DNA vector pD5H8 [**32**] using an In-Fusion HD Cloning Kit (Takara Bio). The final plasmid (pD5H8-MAG) was sequenced by Eurofins Genomics to confirm the correct insertion of the target sequences. Following the transformation of *E. coli* (DH5α), the plasmid DNA (pD5H8-MAG) was isolated and introduced into *T. thermophila*, as described in Section 2.5.


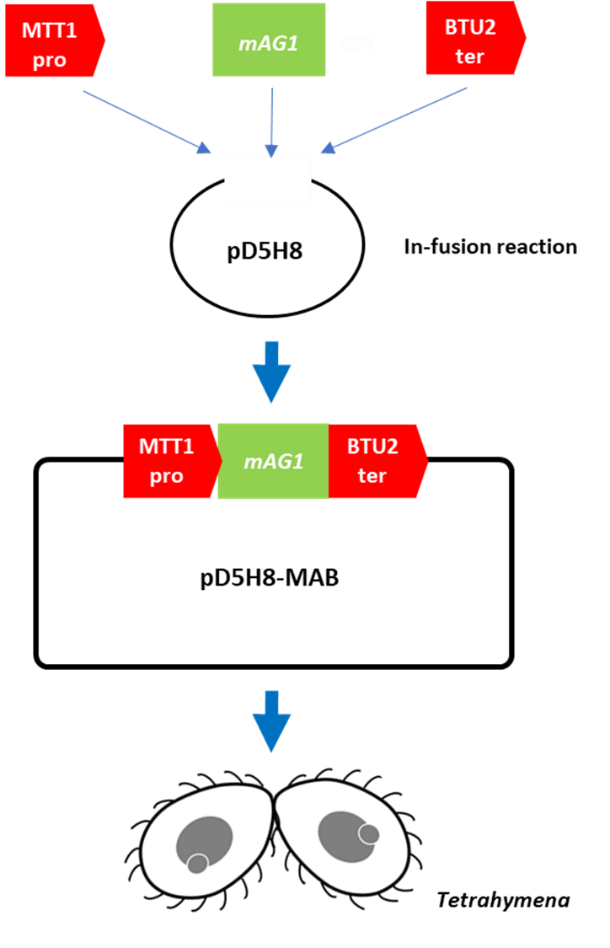


**Fig. S2** Production of pD5H8-MAB plasmid and transgenic *Tetrahymena* cells expressing for r-mAG1.


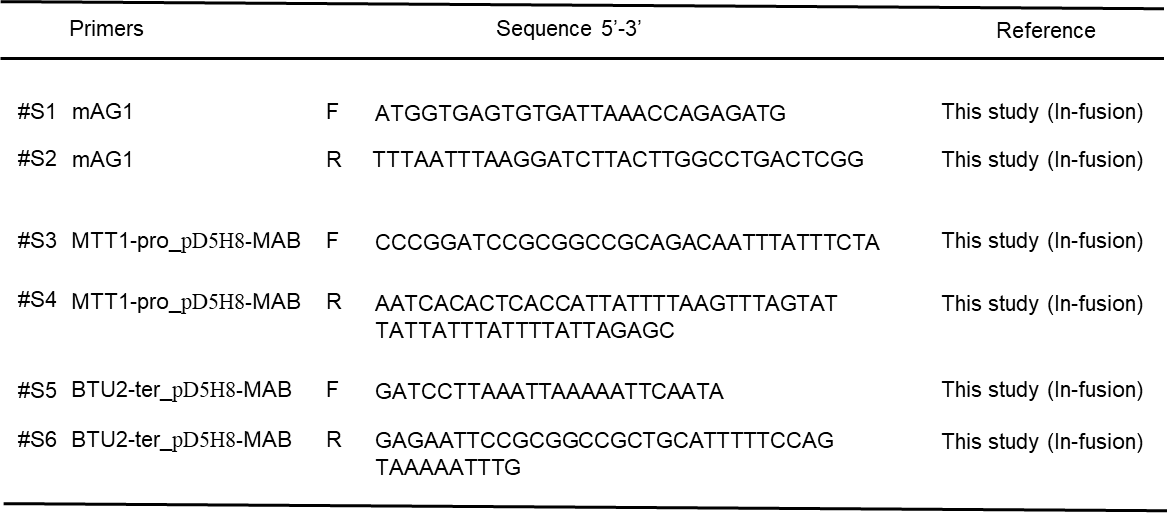
Table S1 Primers for pD5H8-MAB plasmid construction.


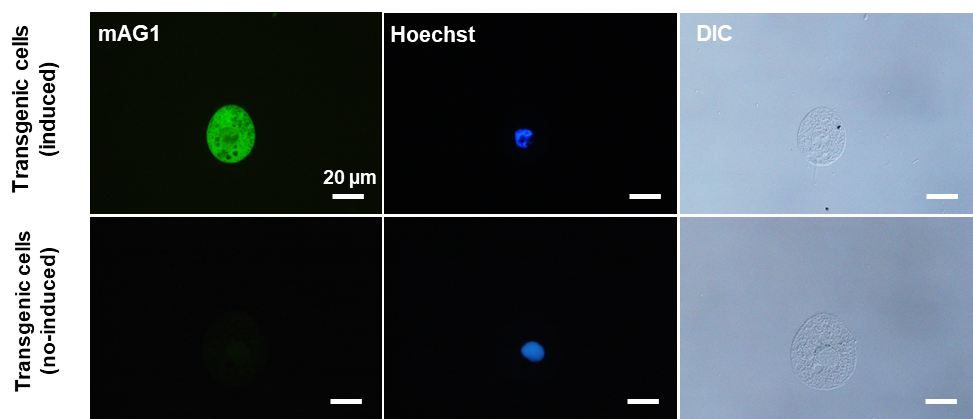


Fig. S3 Immunofluorescence antibody test of transgenic *Tetrahymena* cells (pD5H8- MAB) using the anti-mAG1 antibody. Fluorescence microscopy images of transgenic *Tetrahymena* cells. Scale bar = 20 μm.


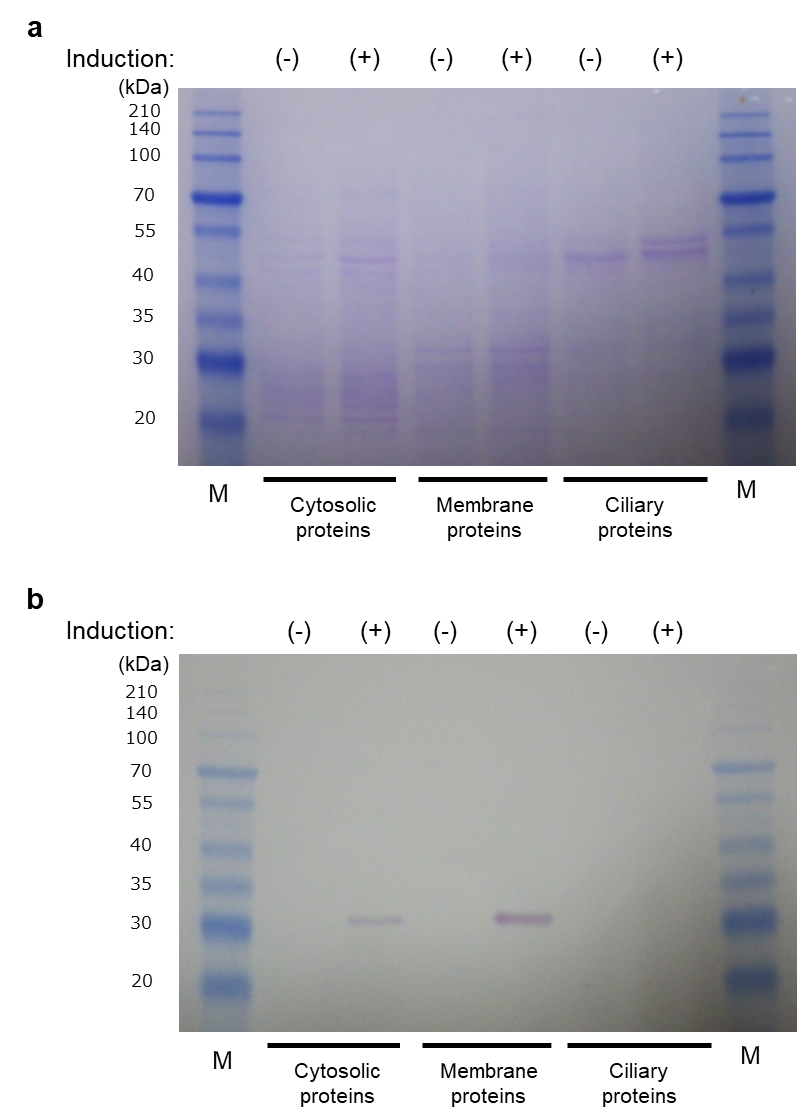


**Fig. S4** Protein expression in transgenic *Tetrahymena* cells (pD5H8- MAB)

(a) SDS-PAGE of transgenic *Tetrahymena* lysates with and without the induction of expression with CdCl_2_. (b) Western blot of the lysates using the anti-mAG1 antibody. M: molecular weight maker.
